# Analysis of two minimally invasive procedures for osteoporotic vertebral compression fractures with intravertebral cleft: a systematic review and meta-analysis

**DOI:** 10.1186/s13018-020-01938-6

**Published:** 2020-09-10

**Authors:** Hongyu Wei, Chunke Dong, Yuting Zhu, Haoning Ma

**Affiliations:** 1 Department of Orthopaedic Surgery, China-Japan Friendship Hospital, 2 Yinghuadong Road, Chaoyang District, Beijing, 100029 China; 2 Beijing University of Chinese Medicine, 11 North Third Ring Road East, Chaoyang District, Beijing, 100029 China; 3 Beijing Tongzhou Integrative Medicine Hospital, 89 Chezhan Road, Tongzhou District, Beijing, 101100 China

**Keywords:** Osteoporotic vertebral compression fractures, Intravertebral cleft, Vertebroplasty, Kyphoplasty, Meta-analysis

## Abstract

**Background:**

A systematic review and meta-analysis to assess the pros and cons of percutaneous vertebroplasty (PVP) versus kyphoplasty (PKP) for osteoporotic vertebral compression fractures (OVCFs) with intravertebral cleft (IVC) including all available evidence from controlled trials.

**Methods:**

Databases including Pubmed, Embase, Cochrane Library, China National Knowledge Infrastructure (CNKI), and Wanfang Data were searched to identify relevant studies comparing PVP and PKP for OVCFs with IVC. The outcomes mainly included visual analog scale (VAS), Oswestry Disability Index (ODI), local kyphotic angle (LKA), rate of vertebral height (VH%), and adverse events.

**Results:**

Nine studies enrolling 688 patients were eligible for meta-analysis. The results indicated no significant differences between the two groups in the short-and long-term VAS, ODI, LKA, or VH% (*P* > 0.05). Compared with PVP, PKP was associated with significantly longer operation time (*P* < 0.05), higher cost (*P* > 0.05), and more injected cement volume (*P* < 0.05). In terms of adverse events, PKP has a lower risk of cement leakage (*P* < 0.05), while with no significant difference in adjacent-level fracture rates (*P* > 0.05)**.**

**Conclusion:**

The two procedures have similar short- and long-term pain relief, functional recovery, local kyphosis correction, and vertebral height maintenance in OVCFs with IVC. PKP is superior to PVP for the injected cement volume, and lower cement leakage rate, however, with longer operation time, more fluoroscopy times, and higher cost. Further randomized controlled trials (RCTs) should be conducted to confirm these results.

## Background

The intravertebral cleft (IVC), which was first described by Maldague in 1978 [1], has long been considered as the result of ischemic necrosis in osteoporotic vertebral compression fractures (OVCFs) [2, 3]. Initial reports dealt exclusively with air-filled IVC, presenting with a transverse, linear, or semilunar radiolucent shadow on conventional radiographs [3, 4]. Then, several reports have also observed variable appearance of IVC in MRI, which depends on whether they are filled with gas or fluid at the time they are imaged [5]. Some scholars propose that this alternating air or fluid phenomenon can be considered indicative of microinstability at the cleft site, which ultimately forms a painful progressive kyphosis due to delayed vertebral collapse, also known as Kummell’s disease [6–8].

Owing to the progression of kyphosis with vertebral collapse and intravertebral instability at the cleft site, fractures with IVC are more susceptible to no response to conservative treatment; thus, surgical intervention may be a better choice [9, 10]. Open surgery has been described to treat IVC, but it is mainly indicated for patients with neurological deficits [11, 12]. Besides, it is inappropriate for most patients with serious comorbidities and severe osteoporosis [13]. Invasive procedures, such as PKP and PVP, widely and successfully used in OVCFs, are better choices for patients with IVC.

Previous studies suggested that PKP displayed better performance in long-term VAS, ODI, LKA, and VH % compared with PVP for OVCFs without IVC [14–17]. During the past two decades, some scholars have tried to use PKP and PVP to treat IVC, but the results are not consistent [18–21]. The pros and cons of the two interventions in treating OVCFs with IVC still need to be investigated. Therefore, we conduct a systematic review and meta-analysis of the available literature to calculate a pooled estimate of the advantages and disadvantages of PKP compared to PVP for single-level OVCFs with IVC.

## Methods

Our meta-analysis was carried out according to the Preferred Reporting Items for Systematic Reviews and Meta-analyses (PRISMA) [22] statement.

### Search strategy

Electronic databases Pubmed, Embase, Cochrane Library, CNKI, and Wanfang Data, were searched for all relevant studies until June 2020. The keywords for the study object included “intravertebral cleft,” “intravertebral vacuum cleft (sign),” “intravertebral pseudarthrosis,” “avascular necrosis,” “vertebral osteonecrosis,” “intraosseous vacuum phenomena,” or “Kummell's disease.” The keywords for the intervention strategy were “vertebroplasty” and “kyphoplasty.” Reference lists of all eligible original and review articles were also screened manually to identify any initially omitted studies. There is no restriction on publication language.

### Inclusion criteria

1. Interventional studies (RCTs) and observational studies (cohort or case-control studies).
2. Studies reported the comparisons between PKP and PVP for patients with single-level OVCFs complicated IVC.
3. Studies reported at least one of the following outcomes: VAS, ODI, LKA, VH%, operation time, injected cement volume, the incidence of cement leakage, and adjacent vertebral fracture.
4. All patients have at least 12-months follow-up period.

### Exclusion criteria

1. Patients suffered from multi-level OVCFs with or without IVC.
2. Pathological fracture due to primary or metastatic tumors, infection, or tuberculosis.
3. Patients complicated with nerve disorder, long-term use of steroidal or nonsteroidal anti-inflammatory drugs, or previous surgery at the diseased vertebra.
4. Non-original articles (case reports, reviews, letters, meta-analyses, and editorials), animal studies, in vitro biomechanical studies, or computational modeling studies.

### Study selection

Two authors (HY.W. and CK.D.) independently screened all titles and abstracts related to the eligibility criteria described above. The full-text of the literature was reviewed thoroughly for the final inclusion. Disagreements were resolved by reaching a consensus with the third author (YT.Z.).

### Data extraction and quality assessment

Baseline data (first author and year of publication, language, study design, sample size, age, gender, and duration of follow-up), intervention, and outcomes were independently extracted in duplicate using a standardized form by two authors (HY.W. and CK.D.). Data in other forms (i.e., median, interquartile range, and mean ± 95% confidence interval (CI)) were converted to mean ± standard deviation (SD) according to the Cochrane Handbook [23]. We extracted data by manual measurements from figures if they were not reported in numbers.

The Cochrane Collaboration’s tool for assessing risk of bias [24] was used to evaluate RCTs’ methodological quality. Each study was judged according to six items: random sequence generation, allocation concealment, blinding of participants and personnel, incomplete outcome data, selective reporting, and other bias. Each piece was classified into three levels: high, unclear, and low risk. The Newcastle-Ottawa-Scale (NOS) [25] was used to assess the methodological quality of cohort or case-control studies on three dimensions: selection (0–4 points), comparability (0–2 points), and the determination of either the exposure or the outcome of interest (0–3 points). The studies with 7–9 points were considered high quality, 5–6 points as moderate quality, and 0–4 as poor quality. The data extraction and quality assessment were independently performed by two authors (HY.W. and CK.D.). The third author (HN.M.) was the adjudicator when no consensus could be achieved.

### Statistical analysis

Our meta-analysis was performed through RevMan v5.3 software (Cochrane Collaboration, Oxford, UK). Continuous data, such as VAS, ODI, LKA, VH%, operation time, and injected cement volume, were expressed as mean ± SD and summarized using the mean difference (MD) or standardized mean difference (SMD) with 95% confidence interval (CI). Risk ratio (RR) with 95% CI was calculated for binary outcome data like cement leakage and adjacent-level fractures. Heterogeneity was tested using the chi-square test and quantified by calculating the *I*^2^ statistics. *P* < 0.1 and *I*^*2*^ > 50% was considered statistical heterogeneity. A random-effects model was used for heterogeneous statistical data. Otherwise, a fixed-effects model was performed. Sensitive analysis or subgroup analysis was used to investigate the source of heterogeneity. Meta-analyses results were also assessed using forest plots, and *P* < 0.05 was considered statistically significant.

## Results

### Literature search and study characteristics

**Fig. 1:**
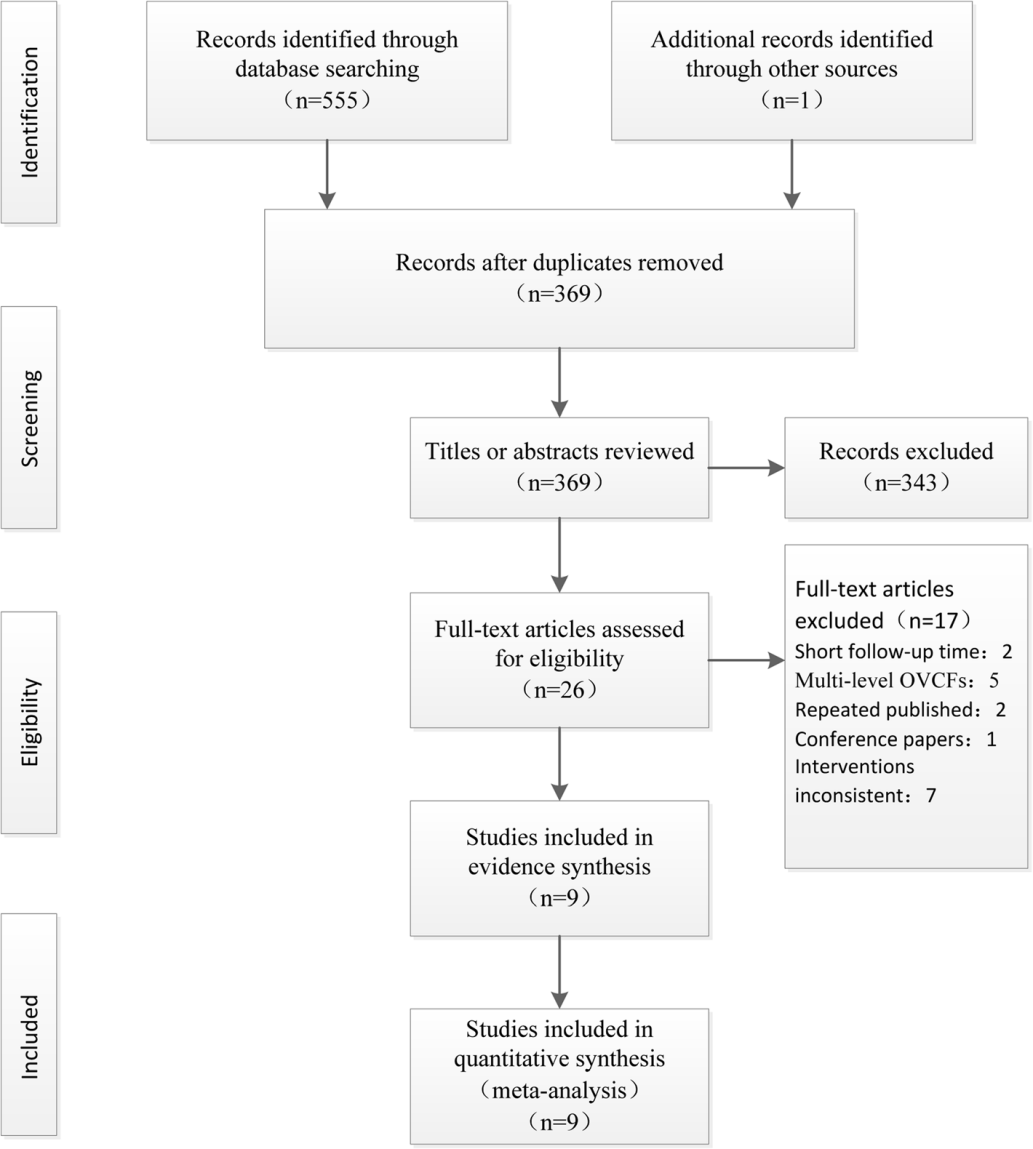
Summary of study selection and exclusion process

In total, 556 citations were identified after an initial systematic search, of which 187 excluded for duplication, and 343 were excluded scrutiny of the titles and abstracts. After reviewing the full text of the remaining 26 studies, we excluded 17 additional articles. Finally, 9 studies [26–34] eventually satisfied the eligibility criteria for meta-analysis, which included one RCT study [26] and eight retrospective cohort studies [27–34]. The results of literature search and study selection process are summarized in Fig. 1. There were a total of 688 patients and 688 vertebral bodies; 378 patients underwent the PVP and the remaining 310 received the PKP. Individual study sample sizes ranged from 35 to 264 patients. The demographic characteristics of the included studies are summarized in Table 1

**Table 1:** Basic characteristics of included studies and NOS scores for the non-RCTs

Study	Language	Study design	Sample size		Age (years)		Gender (M/F)		BMD (*T*-score)		Course of disease (months)		Follow-up (months)	NOS scores
			PVP	PKP	PVP	PKP	PVP	PKP	PVP	PKP	PVP	PKP		
Chang et al. [26]	English	RCT	28	28	75.0 ± 5.8	75.1 ± 5.7	6/22	8/20	− 4.35 ± 0.91	− 4.47 ± 0.89	8.7 ± 3.0	8.4 ± 2.9	33.5 for PVP, 35.2 for PKP	NR
Fang and Yu-Liang [29]	Chinese	RCS	20	20	72.02 ± 4.96	72.43 ± 5.64	5/15	7/13	− 4.7~− 2.6		> 3		10~18	∗∗∗∗∗∗∗
Jiang and Zhang [27]	Chinese	RCS	27	24	74.92 ± 4.97	75.08 ± 4.99	8/19	6/18	− 2.58 ± 0.57	− 2.61 ± 0.63	NR		12	∗∗∗∗∗∗∗
Kong et al. [34]	English	RCS	24	29	70.5 ± 6.4	71.9 ± 7.0	8/16	7/22	NR		4.4 ± 3.5	4.1 ± 3.7	12	∗∗∗∗∗∗∗
Li et al. [33]	Chinese	RCS	151	113	66.2 ± 5.63	68.7 ± 6.49	69/82	41/72	NR		0.93 ± 0.16	1.0 ± 0.22	> 12	∗∗∗∗∗∗
Yu et al. [31]	Chinese	RCS	48	20	74.6(63 ~ 85)	75.9(65 ~ 87)	10/38	4/16	− 4.34 ± 0.94	− 4.35 ± 0.74	0.83	0.79	24	∗∗∗∗∗∗∗
Xing et al. [30]	Chinese	RCS	20	28	67.05 ± 4.03	67.21 ± 4.57	2/18	3/25	NR		NR		> 12	∗∗∗∗∗∗∗
Zhang et al. [32]	English	RCS	38	35	75.58 ± 4.97	73.74 ± 4.35	10/28	9/26	NR		3.76 ± 1.68	4.05 ± 2.01	17.7 for PVP, 17.9 for PKP	∗∗∗∗∗∗∗
Zhang et al. [28]	English	RCS	22	13	72.82 ± 6.99	74.38 ± 5.66	7/15	5/8	− 3.27 ± 0.49	− 3.49 ± 0.62	7.86 ± 4.37	10.15 ± 6.30	22.7 for PVP, 19.3 for PKP	∗∗∗∗∗∗∗

*BMD* bone mineral density, *NOS* Newcastle-Ottawa-Scale, *PVP* percutaneous vertebroplasty, *PKP* percutaneous kyphoplasty, *RCT* randomized controlled trial, *RCS* retrospective cohort study, *M* male, *F* female, *NR* not reported

### Quality assessment

**Fig. 2:**
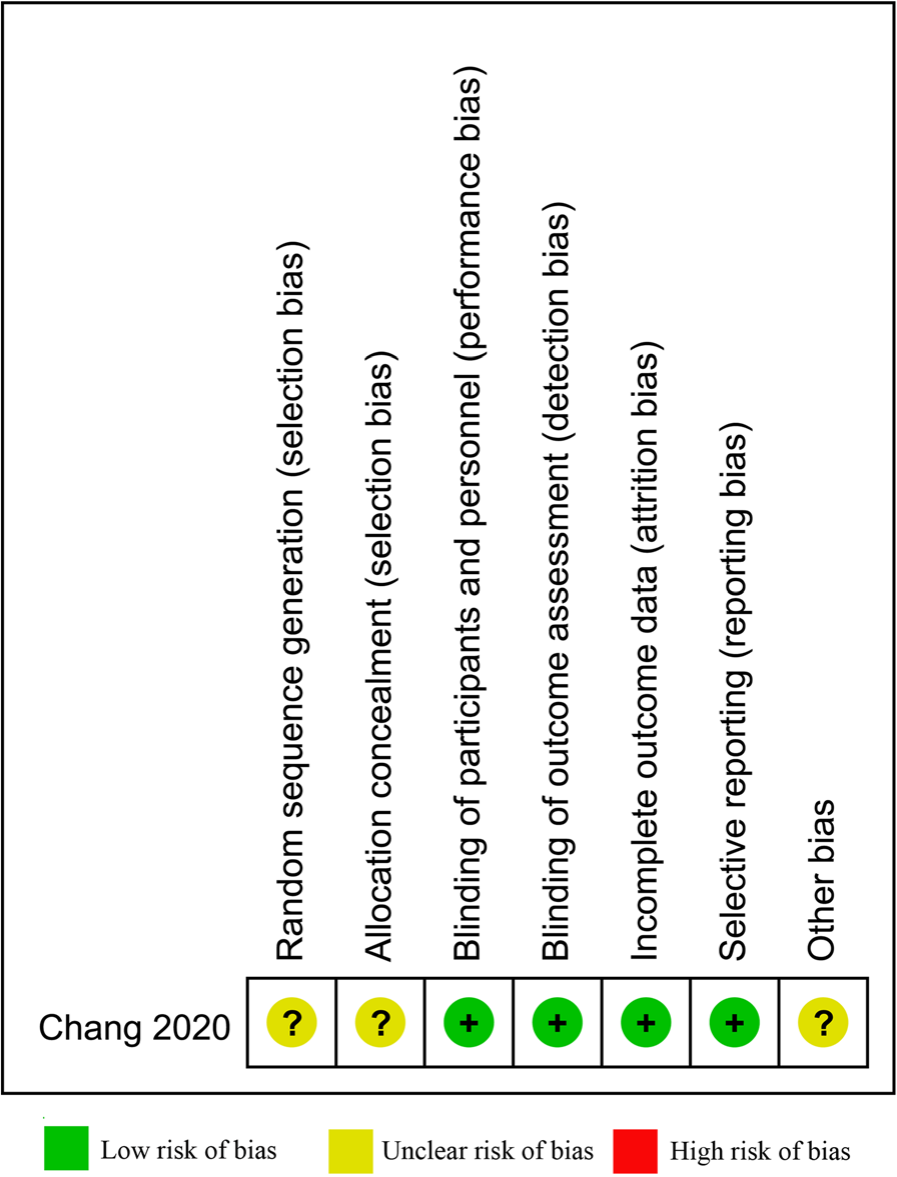
The methodological quality of the RCT

The methodological quality of the included RCT was assessed using Cochrane review criteria, and the result is presented in Fig. 2. In contrast to the RCTs, the non-RCTs used a NOS form. Seven retrospective cohort studies assigned 7 scores were considered high quality, and the remaining one assigned 6 scores was considered moderate quality (Table 1).

### Results of the meta-analysis

#### Pooled analysis of VAS scores

**Fig. 3:**
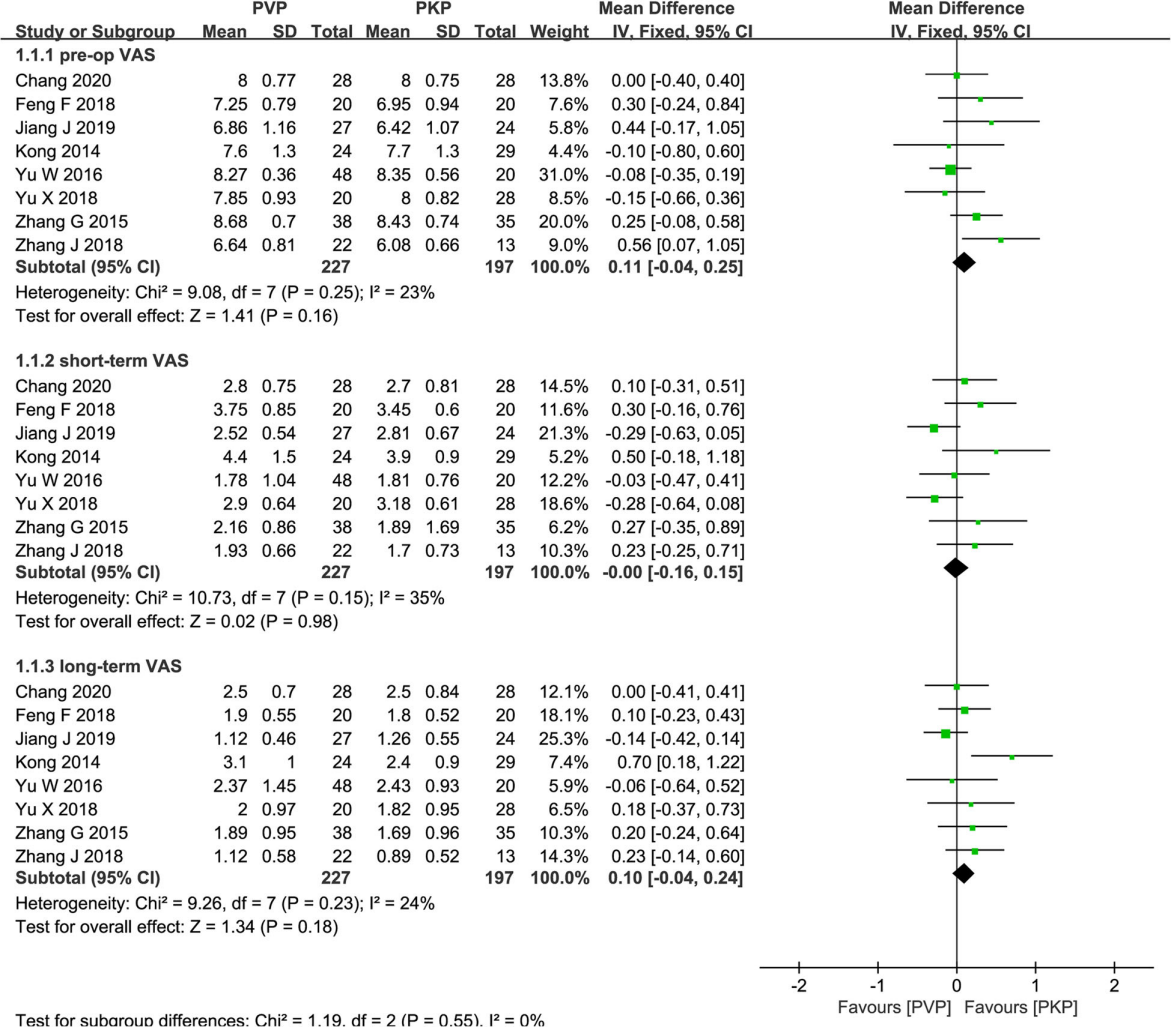
Forest plot of the mean difference (MD) in pre-operation, short-term, and long-term VAS between the PVP and PKP groups

We divided the results into short-term (< 1 month) and long-term (> 1 year). A total of eight studies [26–32, 34] reported short- and long-term VAS. We pooled the outcomes by subgroup analysis, and a fixed-effects model was used because there was no heterogeneity in the short- and long-term VAS values (*P* = 0.15, *I*^2^ = 35%; *P* = 0.23, *I*^2^ = 24%, respectively). The meta-analysis found no significant differences between PVP and PKP for OVCFs with IVC in short-term VAS (MD = − 0.00; 95% CI − 0.16, 0.15; *P* = 0.98; Fig. 3) and long-term VAS (MD = − 0.10; 95% CI − 0.04, 0.24; *P* = 0.18; Fig. 3)

#### Pooled analysis of ODI

**Fig. 4:**
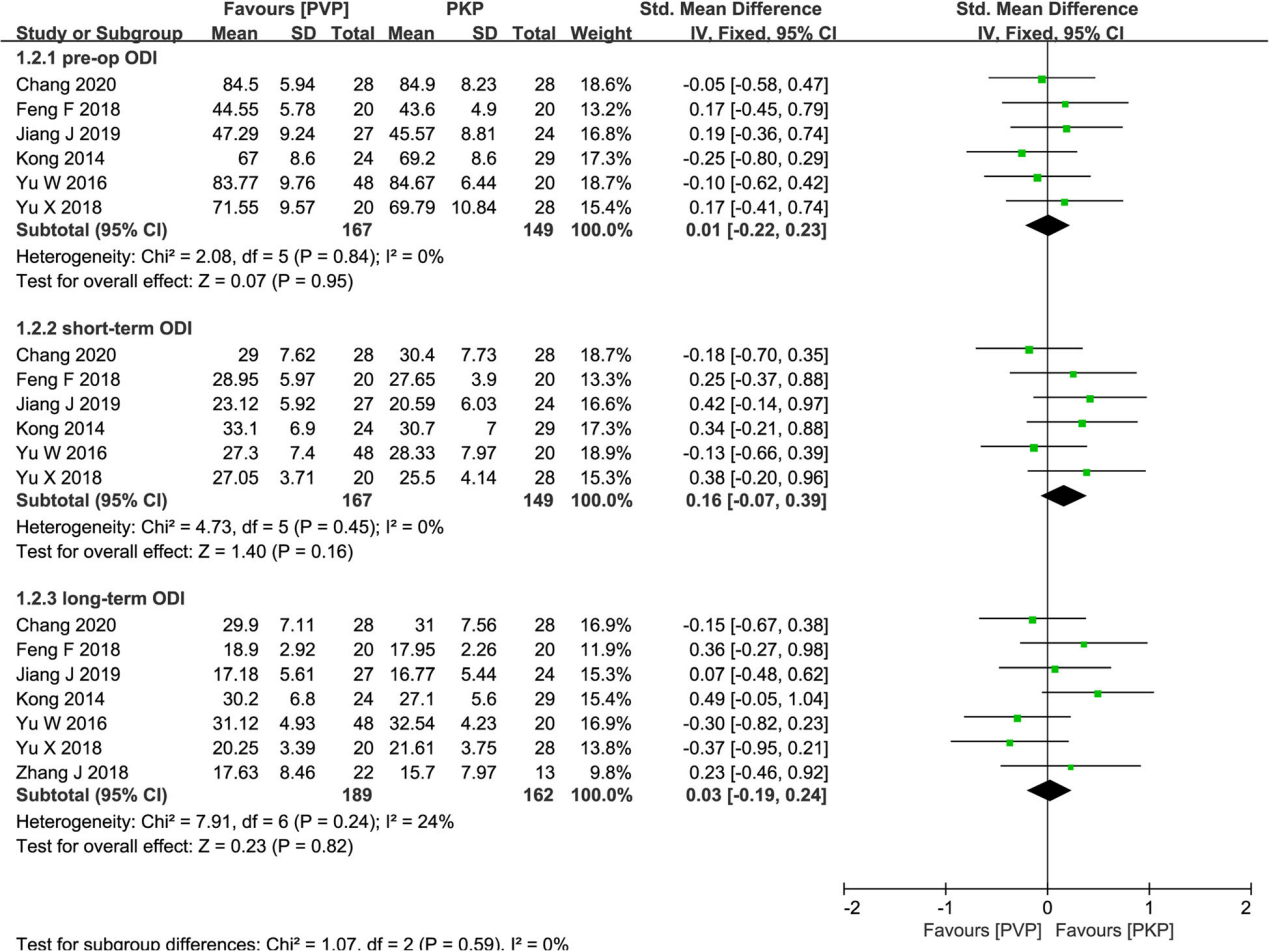
Forest plot of the standardized mean difference (SMD) in pre-operation, short-term, and long-term ODI between the PVP and PKP groups

Six articles [26, 27, 29–31, 34] reported short-term ODI (< 1 month) and seven [26–31, 34] reported long-term ODI (> 1 year). Among them, two studies [27, 29] used ODI scores as the measurement results, and the other studies were measured as ODI%, so SMD with 95% CI was used for summary. Heterogeneous test showed no heterogeneity among the short- and long-term outcomes (*P* = 0.45, *I*^2^ = 0%; *P* = 0.24, *I*^2^ = 24%, respectively). The fixed-effects model showed that there was no significant difference in in short-term ODI (SMD = − 0.16; 95% CI − 0.07, 0.39; *P* = 0.16; Fig. 4) and long-term ODI (SMD = 0.03; 95% CI − 0.19, 0.24; *P* = 0.82; Fig. 4) between the two groups.

#### Pooled analysis of LKA

**Fig. 5:**
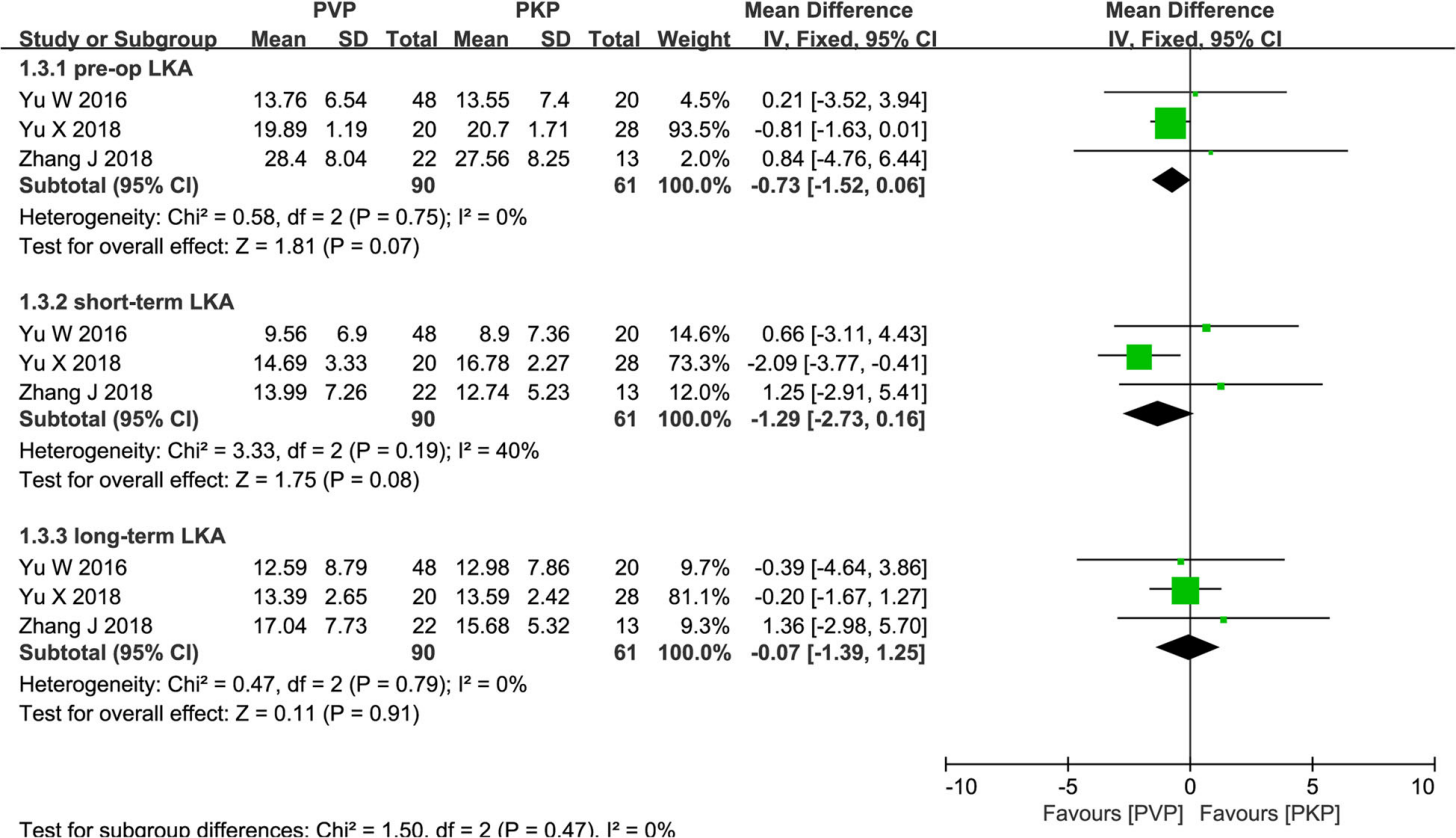
Forest plot of the mean difference (MD) in pre-operation, short-term, and long-term LKA between the PVP and PKP groups

LKA, measured as the angle between the superior endplate of the adjacent upper vertebra and the inferior endplate of the lower vertebra, was reported in three studies [28, 30, 31] including short- and long-term values. The results showed patients who underwent PVP had the similar LKA than patients who underwent PKP in the short-term (MD = − 1.29; 95% CI − 2.73, 0.16; *P* = 0.08; Fig. 5) and long-term follow-up (MD = − 0.07; 95% CI − 1.39, 1.25; *P* = 0.91; Fig. 5) in a fixed-effects model (*P* = 0.19, *I*^2^ = 40%; *P* = 0.79, *I*^2^ = 0%, respectively; Fig. 5).

#### Pooled analysis of VH%

**Fig. 6:**
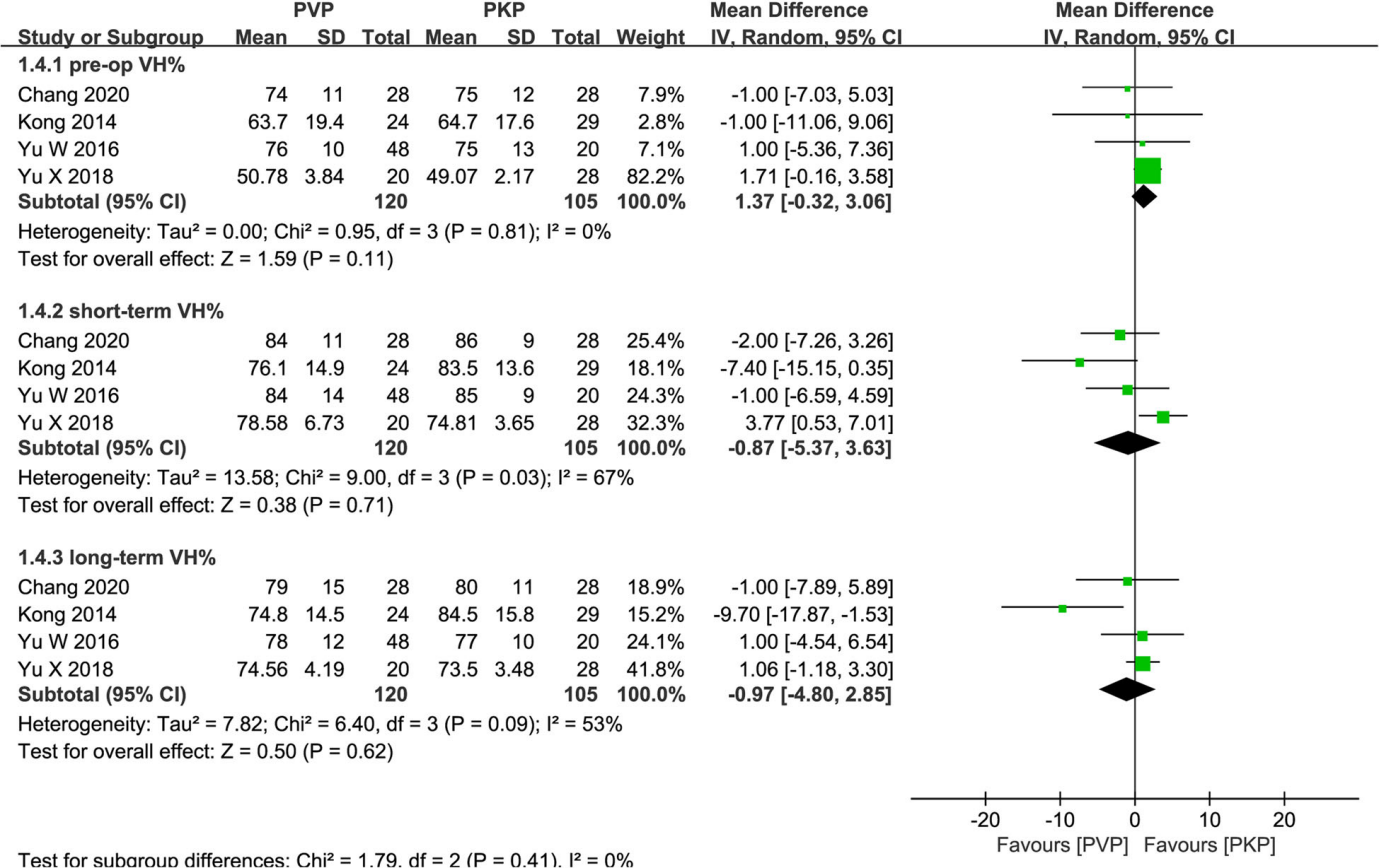
Forest plot of the mean difference (MD) in pre-operation, short-term, and long-term VH% between the PVP and PKP groups

Four studies [26, 30, 31, 34] reported short- and long-term VH%. VH% was measured as the height of the diseased vertebra/the average height of adjacent upper and lower vertebral bodies. The summarized estimate of a random-effects model indicated the absence of significant differences in the short- and long-term VH% between the PVP and PKP group (MD = − 0.87; 95% CI − 5.37, 3.63; *P* = 0.71; MD = − 0.64; 95% CI − 4.67, 3.40; *P* = 0.76; respectively; Fig. 6).

#### Injected cement volume

**Fig. 7:**
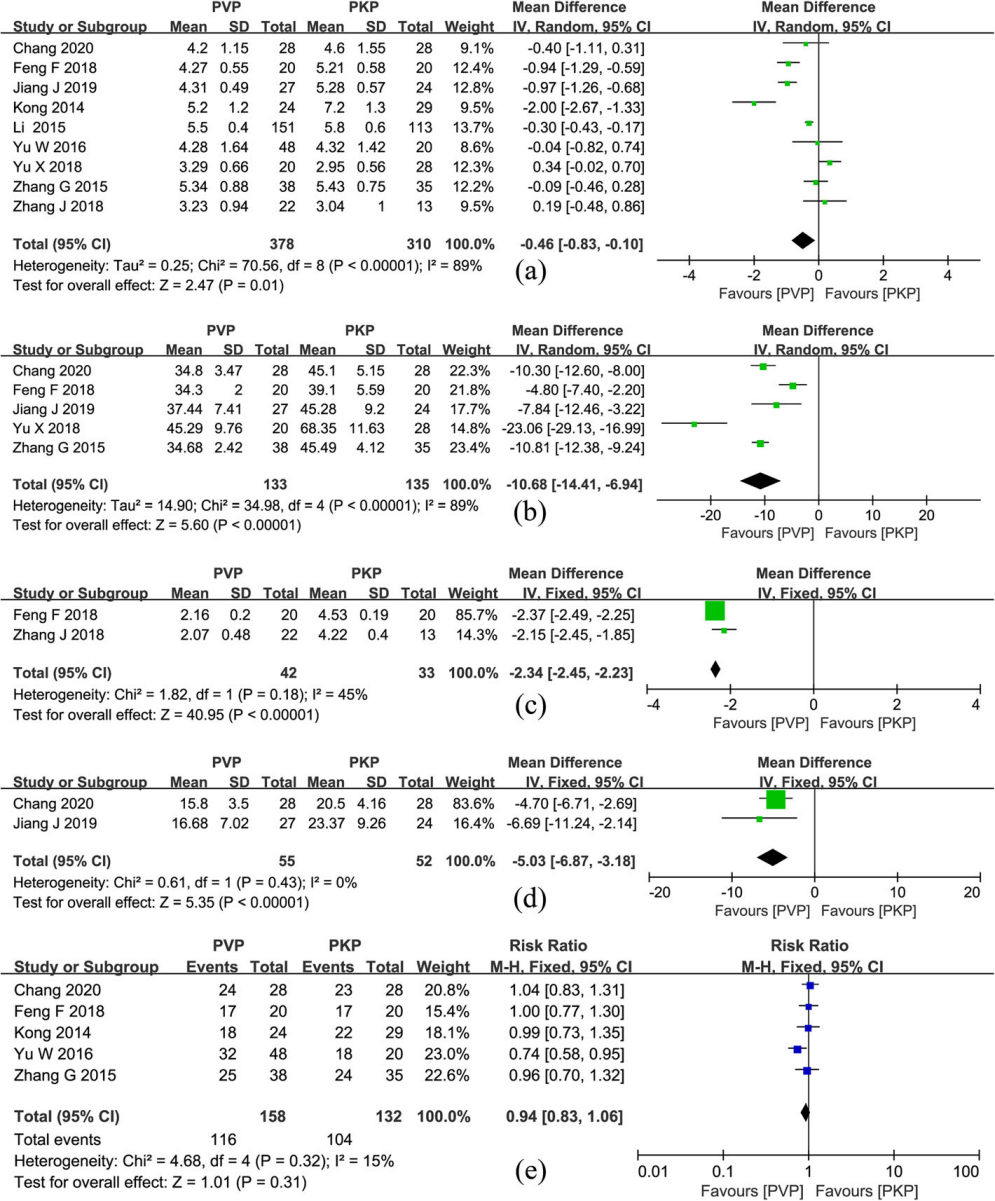
**a** Forest plot of injected cement volume. **b** Forest plot of operation time. **c** Forest plot of operation cost. **d** Forest plot of fluoroscopy times. **e** Forest plot of IVC located in the thoracolumbar

Nine component studies [26–34] provided relevant data, with 378 patients in the PVP group and 310 patients in the PKP group. A random-effects model was used. The PKP group injected more bone cement than PVP group (MD = − 0.46; 95% CI − 0.83, − 0.10; *P* = 0.01), with significant heterogeneity between trials (*P* < 0.00001, *I*^2^ = 89%. Fig. 7a)

#### Operation time

This outcome was described in 5 papers [26–30, 32–34]. The PKP group used more operation time than PVP group (MD = − 10.81; 95% CI − 12.38, − 9.24; *P* < 0.00001. Fig. 7b), The *I*^2^ value attributed 89% variation to heterogeneity; therefore, a random-effects model was used.

#### Operation cost

Three studies [28, 29, 34] reported operation cost, and we ruled out one trial [34] because the premature inclusion of patients may impact the results. The meta-analysis of eventually including studies showed that the operation cost in the PKP group was higher than that in the PVP group (MD = − 2.34; 95% CI − 2.45, − 2.23; *P* < 0.00001. Fig. 7c)

#### Fluoroscopy times

The fluoroscopy times was reported in two studies [26, 27]. A fixed-effects model indicated that the PKP group’s fluoroscopy times was more than the PVP group (MD = − 5.03; 95% CI − 6.87, − 3.18; *P* < 0.00001. Fig. 7d)

#### IVC located in the thoracolumbar (T11–L2)

Five studies [26, 29, 31, 32, 34] described the segmental distribution of lesions in detail. A fixed-effects model showed that the two groups had similar IVC segmental distribution in the thoracolumbar (RR = 0.94; 95% CI 0.83, 1.06; *P* = 0.31. Fig. 7e)

#### Pooled analysis of cement leakage

**Fig. 8:**
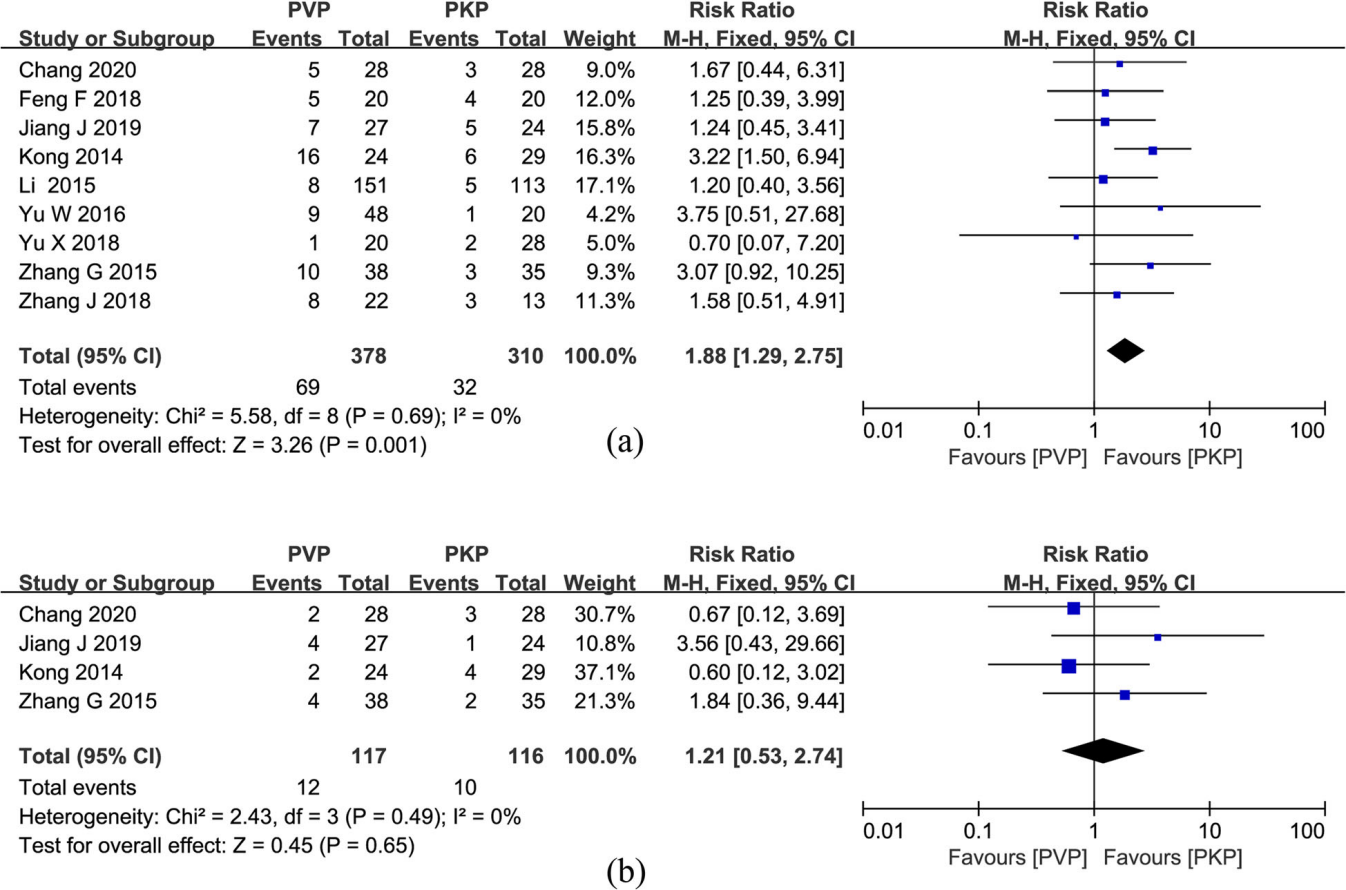
**a** Forest plot of cement leakage. **b** Forest plot of adjacent-level fractures

Relevant data on cement leakage was provided in all studies [26–34], with a total of 688 patients (378 in the PVP group and 310 in the PKP group). The meta-analysis of the 9 studies showed that the PVP group had a significantly higher risk of cement leakage than the PVP group (RR = 1.88; 95% CI 1.29, 2.75; *P* = 0.001; Fig. 8a) in the absence of statistical heterogeneity (*P* = 0.69, *I*^2^ = 0%).

#### Pooled analysis of adjacent-level fractures

Four studies [26, 27, 32, 34] provided data about the risk ratio for adjacent-level fractures. The pooled analysis of a fixed-effects model showed that there was no significant difference between the two interventions in the incidence of adjacent-level fractures (RR = 1.21; 95% CI 0.53, 2.74; *P* = 0.65; Fig. 8b).

### Publication bias

**Fig. 9:**
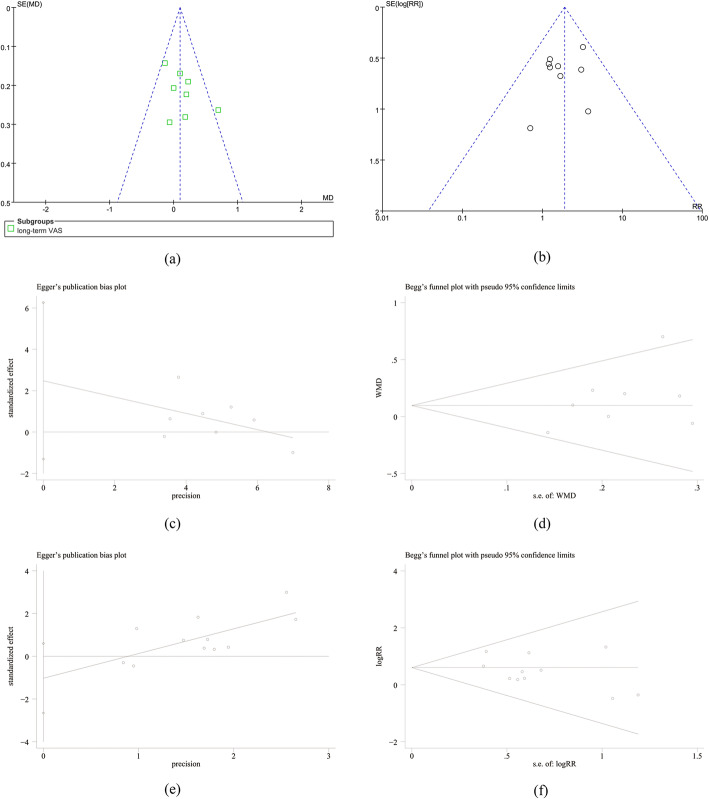
**a**, **b** Funnel plots of long-term VAS and cement leakage. **c**, **d** Egger’s publication bias plot and Begg’s funnel plots of long-term VAS. **e**, **f** Egger’s publication bias plot and Begg’s funnel plots of cement leakage. MD, mean difference; SE, standard error; RR, risk ratio

The review of the funnel plots could not rule out the potential publication bias for long-term VAS (Fig. 9a) and cement leakage (Fig. 9b). The Egger’s and Begg’s tests showed no evidence of publication bias for long-term VAS (*P* = 0.711 and 0.160, respectively; Fig. 9c, d), and cement leakage (*P* = 0.350 and 0.186, respectively; Fig. 9e, f).

### Sensitivity analysis

The sensitivity analysis which was performed by omitting 1 study in each turn investigated the influence of a single study on the overall outcome. The long-term VAS in the PVP group was not significantly different from that in the PKP group when omitting any of the studies except Jiang et al. [27]. The injected cement volume in the PVP group was similar to that in the PKP group when removing one study conducted by Feng et al. [29], Jiang et al. [27], Kong et al. [34], or Li et al. [33]. The other outcomes of sensitivity analysis were not materially differentiated from those of the original analysis.

## Discussion

With the development of imaging technology, IVC, initially, as a rare phenomenon of OVCFs [35], is considered to be a commonly recognized condition at present [36, 37]. Although a consistent pathomechanism has not yet been reached, IVC highly suggests a sign of ischemic posttraumatic vertebral necrosis, known as Kummell’s disease [38]. Instability at the cleft site often leads to pseudarthrosis, which eventually results in severe pain, kyphosis, and nerve injury. Due to insufficient knowledge of the condition and the paucity of literature, specific treatment protocols are limited [39]. Early reports focused on conservative treatment, while more recent preferred surgical interventions for earlier patient ambulation and correction of kyphosis [40]. The two minimally invasive procedures, PVP and PKP, are indicated to eliminate the motion at the cleft site, to maintain the anterior height of the vertebral body, and to relieve pain and are widely used in the treatment of OVCFs with IVC. However, their results were inconsistent, and it is not clear which one could provide better outcomes. Therefore, an evidence base is essential for surgeons to develop a more appropriate scheme. To the best of our knowledge, this is the first comprehensive meta-analysis to evaluate the therapeutic efficacy difference comparing PVP and PKP treatments for single-level OVCFs with IVC.

The dynamic motion within the IVC, expressed by the changes in vertebral body height according to extension and flexion lateral radiographs [41, 42], has been correlated with a high probability of severe pain [43]. VAS and ODI were used to evaluate the pain relief and functional recovery after the operation, respectively. Compared with previous meta-analysis results that PKP was more effective on the VAS and ODI assessments than the PVP in non-IVC OVCFs [44], the pooled results in our article showed no statistical difference between the two groups in short- and long-term follow-up, which indicated they both are effective methods for OVCFs with IVC. Taylor et al. [45] found some evidence of moderate correlations between the change in VAS pain with the change in vertebral height (*r* = 0.62, *P* = 0.184) and change in kyphotic angle (*r* = – 0.68, *P* = 0.09). Several meta-analysis results showed that for non-IVC OVCFs, PKP, via balloon expansion, had a better potential to restore vertebral height and correct kyphotic deformity compared to PVP, which resulted in a better painful and functional improvement in PKP [14, 15, 17, 44]. As compared to our pooled results, the LKA and VH% in the IVC patients have no statistical difference between the two groups during the follow-up period. Five studies [26, 28, 30–32] in our analysis found that patients with IVC could achieve a spontaneous reduction in the hyperextension position without further balloon expansion, which lead to a considerable reduction effect in the two procedures. These studies also revealed that the LKA and AH% in the two groups gradually lost significantly with time, which was consistent with previous findings [18, 46]. However, there was no significant difference in the vertebral recollapse between the two groups due to the similar bone cement distribution in the low pressure IVC region [26, 28].

Need more complex procedures, such as the repeated establishment of expander channel on the pedicle, the PKP calls for more operation time and fluoroscopy times, which is consistent with our statistic findings. Our pooled analysis also demonstrated that the average cost in the PKP group was significantly higher than the PVP group because of the use of a balloon during operation. From the outcomes of our preliminary meta-analysis, the clinical effects had no significant difference; therefore, the cost gap of the two approaches cannot be ignored. In this study, 75.9% of the IVCs were located in the thoracolumbar segment with no significant difference between the two groups, which suggests that the occurrence of IVC may be attributed to the repeated stress activity and high mobility in the thoracolumbar segment [26]. Although the analysis of injected cement volume in the PKP group was more than in the PVP group (MD = − 0.46; 95% CI − 0.83, − 0.10; *P* = 0.01), six studies showed there was no significant difference in their outcomes (*P* < 0.05). The difference may be related to IVC varied locations in the vertebral body [47].

The rate of cement leakage has drawn considerable attention in OVCFs with IVC. The difference in leakage rate between the two procedures was confirmed in our meta-analysis, in which the incidence of cement leakage is significantly higher in the PVP group, at 18.3 % than in the PKP group, at 10.3 %. Possible reasons for the less frequent cement leakage that may be related to balloon expansion could squeeze the surrounding cancellous bone and create a cavity that allows for a more viscous cement to be injected under lower pressure [48, 49]. Concerning the incidence of adjacent-level fractures, there was no significant difference between the PVP and PKP groups (8.9% *vs.*9.8%).

Although not mentioned in our included studies, the refracture in previously cemented vertebrae after PVP or PKP with the incidence of 0.56–2% is a complication that should not be ignored in patients with IVC [50, 51]. Yu et al. [52] had confirmed that IVC might be the most important predisposing factor for recollapse of the augmented vertebrae in OVCFs. Solid lump cement distribution due to the presence of IVC may intercept mechanical interlock with surrounding cancellous bones. Thus, the recollapse in no cemented area is easily developed during daily activity [47]. Once augmented vertebral refracture occurs, it is difficult for surgeons to determine the appropriate treatment. Several studies found that repeated percutaneous vertebroplasty may be a suitable choice considering the risk of complications for elder patients [50, 53].

## Limitations

Several limitations involved in this study should be considered: (1) Our analysis included a number of cohort studies that might result in selective and performance bias due to the absence of random allocation, allocation concealment, and blinding. (2) Heterogeneity may have been caused by poor non-RCT study design, which induced by the unilateral or bilateral surgical technologies used, varying spinal vertebral bodies, bone mineral density, age, gender, follow-up time, and course of disease differences. (3) Publication bias comes from significant conclusions being more easily published, and only one country publications included in this study may aggravate the bias. (4) Finally, given the limited number of the included studies in the analysis, the findings should be confirmed in future research with more relevant RCTs to obtain more reliable and conclusive data.

## Conclusion

Although there is controversy, this systematic review comparing PVP and PKP for treating OVCFs with IVC demonstrates that the two minimally invasive procedures are both safe and efficacious for similar short- and long-term pain relief, functional recovery, local kyphosis correction, and vertebral height maintenance. PKP is superior to PVP for the injected cement volume, and lower cement leakage rate, however, with longer operation time, more fluoroscopy times, and higher cost. Further RCTs should be conducted to confirm these results.

## Data Availability

All data generated or analyzed during this study are included in this published article

## Abbreviations

PVP: Percutaneous vertebroplasty
PKP: Percutaneous kyphoplasty
OVCFs: Osteoporotic vertebral compression fractures
IVC: Intravertebral cleft
CNKI: China National Knowledge Infrastructure
VAS: Visual analog scale
ODI: Oswestry Disability Index
LKA: Local kyphotic angle
VH%: Rate of vertebral height
RCTs: Randomized controlled trials
MD: Mean difference
SMD: Standardized mean difference
CI: Confidence interval
RR: Risk ratio
